# The Binning of Metagenomic Contigs for Microbial Physiology of Mixed Cultures

**DOI:** 10.3389/fmicb.2012.00410

**Published:** 2012-12-05

**Authors:** Marc Strous, Beate Kraft, Regina Bisdorf, Halina E. Tegetmeyer

**Affiliations:** ^1^Microbial Fitness Group, Max Planck Institute for Marine MicrobiologyBremen, Germany; ^2^Institute for Genome Research and Systems Biology, Center for Biotechnology, University of BielefeldBielefeld, Germany

**Keywords:** metagenomics, binning, tetranucleotide frequencies, interpolated Markov models

## Abstract

So far, microbial physiology has dedicated itself mainly to pure cultures. In nature, cross feeding and competition are important aspects of microbial physiology and these can only be addressed by studying complete communities such as enrichment cultures. Metagenomic sequencing is a powerful tool to characterize such mixed cultures. In the analysis of metagenomic data, well established algorithms exist for the assembly of short reads into contigs and for the annotation of predicted genes. However, the binning of the assembled contigs or unassembled reads is still a major bottleneck and required to understand how the overall metabolism is partitioned over different community members. Binning consists of the clustering of contigs or reads that apparently originate from the same source population. In the present study eight metagenomic samples from the same habitat, a laboratory enrichment culture, were sequenced. Each sample contained 13–23 Mb of assembled contigs and up to eight abundant populations. Binning was attempted with existing methods but they were found to produce poor results, were slow, dependent on non-standard platforms or produced errors. A new binning procedure was developed based on multivariate statistics of tetranucleotide frequencies combined with the use of interpolated Markov models. Its performance was evaluated by comparison of the results between samples with BLAST and in comparison to existing algorithms for four publicly available metagenomes and one previously published artificial metagenome. The accuracy of the new approach was comparable or higher than existing methods. Further, it was up to a 100 times faster. It was implemented in Java Swing as a complete open source graphical binning application available for download and further development (http://sourceforge.net/projects/metawatt).

## Introduction

Prokaryotes (Bacteria and Archaea) comprise a significant portion of the living biomass on earth and sustain the geochemical element cycles, a vastly complicated, planetary-scale metabolic network. Prokaryotes form complicated ecological communities consisting of a multitude of species and only a small fraction of these species has been cultivated in the laboratory, studied experimentally and has a known genome sequence. More importantly, these species have been studied in isolation, after a pure culture was obtained. To further refine our understanding of geochemical element cycling it is essential to study the physiology of microbes in their natural context, i.e., the microbial community. Microbial communities can be cultivated in the laboratory under meaningful, near-natural conditions by continuous cultivation of microbial enrichment cultures.

Given such a mixed microbial culture, metagenomics (sequencing and analysis of DNA obtained from complete microbial communities) is a powerful approach to determine both the community composition and the potential physiology of the abundant community members. This way function-identity relationships (e.g., Walsh et al., 2009; Ettwig et al., 2010) can be resolved in a simple and standard way. “Binning” is an essential step in this analysis. Binning can be performed after assembly of raw sequence reads into contigs and consists of the clustering of those contigs that belong together, constitute a (partial) genome of a single population (or of a group of closely related populations). When the sequencing coverage is sufficiently high and when the “microdiversity” is not too high, the resulting bins can be considered provisional whole-genome-sequences of the source populations. The ecological function of those populations can then be investigated, first by genome annotation and subsequently by experiments. Both for assembly and annotation, well developed algorithms and pipelines are available but the binning is still a bottleneck in metagenomic analysis.

Several approaches have been investigated for the binning problem; they can roughly be divided into similarity-based methods, such as BLAST (Huson et al., 2011) and hidden Markov models (Krause et al., 2008), and compositional approaches such as tetranucleotide frequencies (Teeling et al., 2004a,b; McHardy et al., 2006; Chatterji et al., 2007; Bohlin et al., 2008; Diaz et al., 2009; Saeed et al., 2011), interpolated Markov models (IMM; Kelley and Salzberg, 2010) and Markov chain Monte Carlo models (Kislyuk et al., 2009). The advantage of compositional approaches is that they are able to bin contigs with genes that are not homologous to the reference species. This advantage is essential because even closely related species share only a relatively small core genome and the detection of non-homologous genes in related species is the essence of unraveling new function-identity relationships. The advantage of similarity-based methods is that these approaches are very robust – given a contig of sufficient length they generally provide a clear indication about the (approximate) taxonomic position of the source population. Therefore, without a similarity-based method, it is impossible to evaluate the binning results obtained by a compositional algorithm, except with artificial datasets (e.g., FAMeS; Mavromatis et al., 2007). In practice, a complete binning procedure should therefore consist of a combination of similarity and composition based methods.

Compositional approaches can further be subdivided into supervised (i.e., comparison of the metagenomic contigs to existing genome data) and unsupervised (comparison of the metagenomic contigs only to each other) methods. Given the facts that (a) microbial diversity is vast and (b) relatively few reference genome sequences are available, an unsupervised method is usually essential to prime the binning process; supervised methods do not perform well when no closely related organism is available to train the models. Once bins have been primed with an unsupervised method, models of different types can be trained on the primed bins and the binning can be completed. Such two-step procedures were recently shown to be promising (Kelley and Salzberg, 2010; Saeed et al., 2011).

In the present study we analyzed microbial communities growing in continuous culture in the laboratory. These communities were of medium complexity (up to eight “binnable” populations). Our metagenomic samples contained 4–10 million 50–150 basepairs reads (Illumina) and these were first assembled into contigs. Assembly yielded contigs of reasonable size (longest contigs between 30 and 200 kb). For the binning of these contigs, we developed the new integrated binning procedure that is the topic of this paper. It is similar to the two-step approach described by Kelley and Salzberg (2010) and Saeed et al. (2011) but uses a newly developed, ultrafast algorithm based on multivariate statistics of tetranucleotide frequencies for the priming of the bins.

Compared to previous methods the new unsupervised priming algorithm is very fast (seconds) and does not require an estimate for the number of binnable populations. Further, for each of the produced bins, a taxonomic signature is calculated with a similarity-based approach (BLAST). By inspection of this signature in combination with sequencing coverage information, promising bins can be identified and used to train IMM. These models are then used for final binning.

With this procedure, eight populations from our community could be binned with high apparent accuracy (>90% at the genus level, >96% at the family level). The general performance of our procedure was further evaluated by comparison to two existing methods (Kelley and Salzberg, 2010; Saeed et al., 2011) for four publicly available metagenomes and one previously published artificial metagenome (Table 1). Our evaluation showed that the new approach is much faster and achieves better or comparable accuracy. It was implemented in a stand-alone graphical interactive binning environment, the “Metawatt binner” that is available for download, use, and further development.

**Table 1:** **Performance (time, T, minutes, recall, R, percentage, accuracy, A, percentage) of the presented binning approach (Metawatt) for five publicly available metagenomes in comparison to two published two-step compositional binners: SCIMM (Kelley and Salzberg, 2010) and 2Tbinner (Saeed et al., 2011)**.

Metagenome	Total (Mb)	Contigs	SCIMM			2Tbinner			Metawatt*		
			T	R	A	T	R	A	T*	R	A
Acid mine drainage	11.2	1703	22	79.2	72.3	190	**	**	1	80.4	82.8
*Olavius* symbionts	22.3	868	34	78.2	76.3	371	**	**	1	77.0	88.4
EBPR	24.4	11188	30	83.8	74.5	36	39.3	98.4	3	93.3	81.3
Whalefall bone	28.9	26232	45			24	**	**	4		
SimBG	39	40000	53	77.6	75.6	33	4.5	90	7	91.6	92.6

*Time includes tetranucleotide and IMM training and binning, but not the evaluation by BLAST.

**The *R* script produced an error and/or no meaningful bins were generated.

## Materials and Methods

### Samples and metagenomic sequencing

Eight samples were taken from a microbial enrichment in continuous culture, inoculated with sediment from the Janssand tidal flat in the German Wadden Sea (N 53.73518; E 07.69912). DNA was extracted. Barcoded Illumina TruSeq libraries were generated and sequencing was performed (together with four further libraries of a different study) on one flow cell lane of an Illumina Genome Analyser GA IIx instrument, in a 2 × 150 cycles paired end run. Reads were submitted to the Short Read Archive (SRA, accession number SRP012152) and assembled contigs (see below) to the Whole Genome Shotgun repository (WGS, accession numbers SUB086333, SUB122313, SUB122314, SUB122316, SUB122317, SUB122318, SUB122319. SUB122321).

### Assembly

Assembly was performed with MetaVelvet-v0.3^1^. After quality trimming (sliding window approach: window length 15 basepairs, within this window quality value of at least 99%, minimal read length after trimming 25 basepairs) between 4,414,212 and 9,986,877 reads (392,800,534–901,487,996 bases) per sequenced library were assembled. Assembled contigs were submitted to WGS (see above). An overview of the assembly results is presented in Table 2.

**Table 2:** **Assembly results of the eight sequenced metagenomes**.

	1	2	3	4	5	6	7	8
Number of reads (millions)	4.8	7.6	4.4	8.2	5.6	5.7	**10**	5.2
Total sequence data in reads (Mb)	474	687	424	751	550	537	**901**	393
Number of contigs (thousands)	16	6.3	40	7.6	8.9	**52.6**	5	19.3
Total sequence data in contigs (Mb)	15.3	13.7	20.4	15.7	13.4	**23.3**	13	13.3
Longest contig (kb)	**182**	**182**	77	167	145	37	177	93
N50 contig length (kb)	2.7	7.7	1	5.2	6.8	0.8	**26.3**	1.8
K-mer size for assembly	51	51	51	61	61	61	61	51

### Ultrafast unsupervised binning based on tetranucleotide composition

There exist 256 (4^4^) different tetranucleotides. However, when we assume that both DNA strands are sampled equally, the reverse complements of every tetranucleotide become redundant and 136 non-redundant tetranucleotide pairs remain. (The number of actual degrees of freedom is lower, 103, see Kislyuk et al., 2009). Each of these remaining pairs consists of the tetranucleotide itself and its reverse complement. The frequencies of the 136 different non-redundant tetranucleotide pairs were calculated for each contig, normalized to contig length, and the composition of each contig was represented as a 136-dimensional vector. The normalized frequency of tetranucleotide *x* in contig *y* was calculated as follows:

$$\text{frequency}\left(x\right)=\frac{\text{occurrence}\left(x\text{ in }y\right)}{\begin{array}{c} \text{total number of tetranucleotides}\\ \text{matched in }y*\;136 \end{array}}$$

After the multiplication by 136 and with a GC content of 50%, average frequencies correspond to a value of 1.

**Figure 1:**
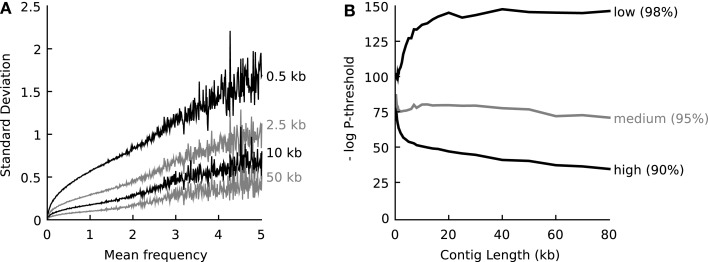
**(A)** When populations of DNA fragments of defined length were sampled from a source genome, an empirical relationship was observed between the mean frequency, the frequency of any tetranucleotide in the source genome, and the standard deviation in the frequency of that tetranucleotide observed in the sample populations. The relationship is shown for four different lengths of DNA fragments and at each mean frequency the average standard deviation is shown. For an explanation on the calculation of the frequencies, see main text. **(B)** Empirical relationship that defines the probability threshold as a function of DNA fragment length at high (90% recall), medium (95% recall) and low (98% recall) confidence. The relationships were determined empirically by sampling 794 representative reference genomes (*n* = 100 for 37 different DNA fragment lengths between 0.3 and 100 kb).

One-hundred artificial contigs of distinct lengths (0.3, 0.4, 0.5, 0.6, 0.7, 0.8, 0.9, 1.0, 1.1, 1.2, 1.3, 1.4, 1.5, 1.75, 2, 2.25, 2.5, 2.75, 3, 4, 5, 6, 7, 8, 10, 12, 14, 16, 18, 20, 25, 30, 40, 50, 60, 70, 80, 100 kb) were sampled from each of the 794 prokaryotic whole-genome-sequences representing the known biodiversity (one genome per genus, Table S1 in Supplementary Material). Tetranucleotide vectors were calculated for each reference genome as a whole (the mean vector) and for each artificial contig (sample vectors). Next, at each contig length *l*, the standard deviation of the tetranucleotide frequencies observed in the population of sample contigs (*s*) was plotted against the mean frequency observed in the source genome (*m*). Figure 1A displays plots for four different values of *l*.

Figure 1 shows that independent of the nature of the tetranucleotide and independent of the source genome, *s* can be predicted when *m* and *l* are known.

We attempted to describe the observed empirical relationship *s* = *f (m, l)* as a formal mathematical function, but no satisfying function was found that accurately described the relationship for all relevant values of *m* and *l*. Therefore, the observed empirical relationship *s* = *f (m, l)* was interpolated into a lookup table to be able to estimate the standard deviation based on the mean frequency and the contig length.

With this lookup table [*s* = f (*m, l*)] it was now possible to estimate the multivariate probability (P) that a contig (length *l*) of unknown origin belonged to any source organism, given the observed tetranucleotide frequency vector *v* for the contig and a known or estimated mean tetranucleotide vector *m* of the source organism:

$$P=\prod_{x=1.136}\frac{1}{\surd 2\pi s_{x}}e^{-\frac{1}{2}\frac{{\left(v_{x}-m_{x}\right)}^{2}}{s_{x}^{2}}}$$

During binning *m*, the mean vector of the “source organism” is estimated as described below.

Next, the artificial contigs were used to determine empirical threshold values for *P* that could be used by the algorithm to decide whether the unknown contig belonged to the source organism or not (Figure 1B). Three thresholds were defined, a high confidence threshold that only accepted 90% of the artificial contigs belonging to a given organism, a medium confidence threshold that accepted 95% of the artificial contigs and a low confidence threshold that accepted 98% of the artificial contigs. It was found empirically that the threshold value for *P* depended on the length *l* of the unknown contig, as shown in Figure 1B. Again, this function was interpolated into a lookup table with *P* threshold = *f*(90, 95, or 98%), *l*).

### Using the relationships of figure 1, the binning now proceeded as follows

First the contigs were sorted by length and tetranucleotide vectors were calculated for all contigs. The longest contig was processed first and a bin was created for this contig. Next, contigs were processed one by one, from long to short, and for each existing bin, the probability that the contig belonged to the bin was calculated. Because the probability value *P* only decreases as more dimensions are analyzed, the comparison was aborted once it fell below the threshold. When no bin could be found for the new contig (all *P* values below *P* threshold), it was used to seed a new bin. Otherwise, the contig was joined with the most probable bin (highest *P*) and a new mean tetranucleotide vector was calculated for that bin (vectors were weighed by contig length).

In this comparison, it was necessary to assume that the tetranucleotide vector of any existing bin approximated the mean vector of its source genome. This approximation would be better for longer contigs, and this was the reason why the contigs were first sorted. For short contigs the approximation would not be valid and therefore, no new bins were seeded with contigs less than 1000 basepairs long. The entire procedure was performed three times, once for every confidence level (low, medium, and high, Figure 1B). Less than 1 min was required to complete each of these calculations with a i5 M430 processor (2.27 GHz) for all datasets (up to 39 Mb of assembled contigs, see Table 1).

### Analysis of bin taxonomic compositions with BLAST

First every contig was fragmented into 500 basepair pieces. BLAST (Camacho et al., 2009) was used to compare these pieces to a database with the 794 prokaryotic whole-genome-sequences representing the known biodiversity (one genome per genus, Table S1 in Supplementary Material). Hits of >200 basepairs length and with at least 25% nucleotide identity were used to create a taxonomic profile for each contig. The profile consisted of five taxonomic ranks (phylum, class, order, family, genus). At each rank the taxon with the most hits was recorded together with the number of hits to this taxon and the median *e*-value of the hits. After binning, the contig profiles were added and averaged to calculate a taxonomic profile for the bin as a whole. This profile was displayed as a pie diagram; see Figure 3 for examples.

### Calculation of sequencing coverage

Next to taxonomic composition, the sequencing coverage constitutes a second, independent criterium to evaluate binning success. Contigs that belong to the same source population should have similar coverage, whereas different source populations can have different coverages (dependent on the relative abundances, chromosome copy number, and DNA extraction efficiency). Coverage was parsed from the header line of the fasta output produced by the assembler or estimated for each contig from the average read length of the sequencing run and the number of source reads for the contig parsed from the header line of the contig fasta file produced by the assembler. The regular expressions used for the parsing of coverage or number of reads were “cov[a–z]*?[=_]([\\d\\.]+)” and “numreads = (\\d+).”

### Interpolated markov modeling

After inspection of the unsupervised binning results for all samples, good bins were selected for the final binning step. “Good bins” were bins with relatively long contigs, a consistent taxonomic profile and a equally distributed sequencing coverage (decision made by the scientist). IMM were created with the program “build-icm” from the Glimmer package (Delcher et al., 2007). The models were used to score all contigs of all samples with the program “simple-score” from the Glimmer package, used with the −N option (no negative model). For each contig, the scores were compared for each model and the contig was binned to the model with the highest score.

### Evaluation of binning accuracy with publicly available metagenomes

Four publicly available metagenomes were selected for evaluation: a metagenome sampled from acid mine drainage (Tyson et al., 2004, accession numbers AADL01000110.1-AADL01001068.1, CH003545.1-CH004435.1, DS995259.1-DS995275.1), one obtained from enhanced biological phospate removing (EBPR) sludge (Martin et al., 2006, accession numbers AATN01000001.1-AATN01011188.1), one from an *Olavius algarvensis* microbial symbiont community (Woyke et al., 2006, accession numbers AASZ00000000.1, DS021108.1-DS022223.1), and one from an Antarctic whale fall bone (Tringe et al., 2005, accession numbers AAGA01000001.1-AAGA01026232.1). In addition, an artificial metagenome was used (SimBG, Saeed et al., 2011). All five metagenomes were also used for evaluation by Saeed et al. (2011). Additional information is provided in Table 1. For evaluation of the real metagenomes, we assumed that the annotations provided by the authors of the original studies were correct. In the EBPR case no annotations were provided, so we used the published genome of *Candidatus “Accumulibacter phosphatis”* as the reference. After binning, a bin was assigned to each population and the accuracy was calculated as the number of correctly binned nucleotides divided by the total number of nucleotides in the bin (×100%). Recall was calculated as the number of nucleotides of the source organism assigned to the bin divided by the total number of nucleotides of the source organism present in the metagenome (×100%). For the Whale Fall metagenome, evaluation of accuracy and recall was impossible, as binning was reported to be unsuccessful by the authors. The results (accuracy, recall, and computation time) were compared to two comparable previously published state of the art *de novo* compositional binners (Kelley and Salzberg, 2010; Saeed et al., 2011). For SCIMM (Kelley and Salzberg, 2010), bins were seeded with a single trial of Likely Bin and the algorithm was run multiple times with different estimates for the number of populations. In Table 1 only the results for the optimal choice are shown. 2T binner was run with the default options.

### Implementation

The procedure was implemented in java as a Swing application that has been tested on Linux (64 bit). A graphical user interface was necessary because our method depends on an important choice by scientist: which tetranucleotide bins should be used to train IMM models? For this reason, visualization of the binning results is important. The application is freely (Academic Free License) available for download and further development at http://sourceforge.net/projects/metawatt. It depends on BLAST (Camacho et al., 2009), glimmer (Delcher et al., 2007) and the batik library^2^ for exporting structured vector graphics (SVG). For evaluation of binning results a BLAST library of sequenced genomes and a taxonomy of these genomes is necessary. Metawatt can generate these files automatically when it is provided with the genbank files of all reference organisms (downloadable from http://ftp.ncbi.nlm.nih.gov/genomes/Bacteria/all.gbk.tar.gz).

## Results

When we inspected multivariate distributions of tetranucleotide frequencies of artificial DNA fragments sampled from reference genomes we observed that for all organisms these distributions can be approximated by a single Gaussian function characterized by a generally valid empirical relationship between the mean frequency of any tetranucleotide in a genome, the standard deviation of the observed frequency in DNA fragments sampled from this genome and the fragment length. See materials and methods and Figure 1A for details. Given a DNA fragment of known length and tetranucleotide composition, the relationship can be used to calculate a probability that the fragment belongs to a source genome with known or estimated mean tetranucleotide composition. Further analysis of calculated probabilities for artificially sampled DNA fragments enabled the definition of a threshold for the probability value that could be used to determine whether a DNA fragment most likely belongs to a source genome or not. Figure 1B shows the empirical relationship between the DNA fragment length and the threshold probability at 90, 95, and 98% recall. See section “Materials and Methods” for details.

The two empirical relationships shown in Figures 1A,B enabled us to rapidly calculate whether a metagenomic contig should be binned together with another contig. Apart from the contig sequences themselves this calculation made use of only a single parameter – the confidence value (90, 95, or 98% recall). It depended on only a single assumption: that the average tetranucleotide composition of the two contigs under consideration approximated the composition of the source genome. The validity of this assumption obviously depended on contig length; the longer the contigs, the better their tetranucleotide composition would approximate that of their source genome. For this reason, contigs were sorted by length before binning.

**Figure 2:**
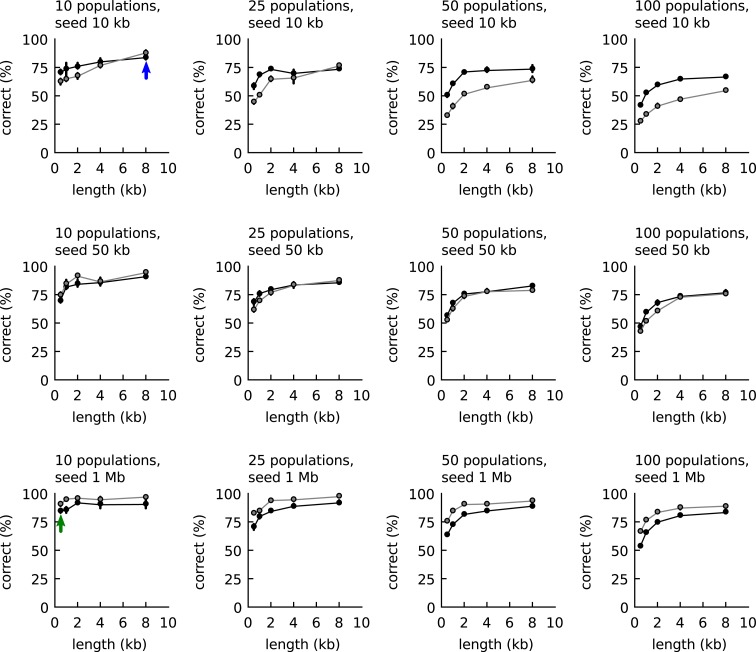
**Classification accuracy with the empirical relationships of Figure 1 (black line, closed symbols) and Interpolated Markov models (gray lines and symbols)**. The accuracy was calculated as function of community complexity (10, 25, 50, and 100 populations), length of the long DNA fragments used for training (10, 50, and 1000 kb, one for each population), and length of the short DNA fragment to be classified (100 fragments, 0.5–8 kb). Each point is the average of three communities randomly sampled from 794 reference genomes. For the explanation of the blue and green arrow, see main text.

We first investigated the possibility to use the two empirical relationships of Figure 1 to classify DNA fragments. The classification accuracy was compared to the accuracy obtained with IMM as follows: first artificial communities were created from reference organisms randomly sampled from 794 available whole-genome-sequences of different genera (Table S1 in Supplementary Material; 10, 25, 50, and 100 species per community). For each species three artificial long DNA fragments were created (10, 50, or 1000 kb) and also five groups of 100 artificial short DNA fragments (500, 1000, 2000, 4000, and 8000 basepairs length). Next, all short DNA fragments were classified based on comparisons with one of the long fragments: either the tetranucleotide frequencies were compared as explained above or an IMM was trained with the long fragment. The classification accuracies are shown in Figure 2. The figure shows that when the DNA fragment used for training was longer than 50 kb, IMMs outperformed our algorithm for classification. With shorter fragments, the tetranucleotide classifier outperformed IMMs.

A metagenomic binner was now created that made use of the tetranucleotide classifier. Binning started by seeding the first bin with the longest contig. Next, the remaining contigs were processed one by one in order of decreasing size. Each contig was binned to the bin that yielded the highest probability value, or when no probability was above the threshold, a new bin was created. When a contig was joined into an existing bin, a new mean frequency vector was calculated for the bin. And so on. With growing bin sizes (and few misbinnings), the mean vectors of the bins would approach those of the source genomes. The two arrows in Figure 2 provide an indication of the level of accuracy achieved during binning. In the initial stages with small bin sizes, the estimate for the tetranucleotide composition of the source genome is poor, but the binned contigs are long (blue arrow) and the accuracy is around 80%. Toward the end, the estimates for the tetranucleotide composition of the source genomes are much better (large bin sizes) but the binned contigs are smaller (green arrow) and the accuracy is also around 80%.

The procedure allowed us to bin a metagenome without an *a priori* estimate for the number of populations that might be binnable, as is necessary for some other algorithms (e.g., Kislyuk et al., 2009). As expected, the binning algorithm was very fast (seconds for all tested metagenomes, up to 39 Mb, see Table 1). We found that no general rule existed as to what confidence (90, 95, or 98% recall) was best for the calculation of the threshold values. In all cases, the application of a high confidence threshold (e.g., 90%) led to a larger number of bins. In some cases, this was justified. In other cases it was not and populations that were binned into a single bin at lower confidence were distributed over multiple bins at higher confidence. For this reason, binning was always performed three times, once for each confidence value.

To evaluate the binning results, a taxonomic profile was created for each bin and its sequencing coverage was calculated (as explained in the Materials and Methods section). The taxonomic profiles and coverage distribution of the bins produced at all three levels of confidence were now inspected. Bins with a consistent taxonomic profile and homogeneous sequencing coverage were selected and used to train IMM, one for every selected bin. These models were then used to rebin all contigs in a final binning step. As shown above, IMMs outperformed our tetranucleotide-based algorithm when much sequence information was available.

The performance of the binner was evaluated with four publicly available metagenomes and one artificial metagenome (Table 1). For comparison the binning was also performed with two other state of the art two-step compositional binners (Kelley and Salzberg, 2010; Saeed et al., 2011). It appeared that our binning procedure was up to a 100 times faster and the accuracy was comparable or better. The overall binning procedure completed in 7 min for the largest metagenome tested (39 Mb). The binning results presented quantitatively in Table 1 are briefly described qualitatively below.

The SimBG artificial metagenome contains contigs belonging to eight different bacteria and one artificial “fake” bacterium (Saeed et al., 2011). The binner made very few errors, except in case of *B. halodurans*. Its contigs were binned only at 70.2% accuracy because of the misbinning of some *P. marinus* and *D. hafniense* contigs.

The EBPR metagenome (Martin et al., 2006) contains contigs belonging to “*Candidatus Accumulibacter phosphatis”* and several side populations. After IMM binning, the contigs belonging to *A. phosphatis* were binned at excellent recall but at relatively low accuracy. Tetranucleotide binning actually yielded some bins with very high accuracy (97.4%) but in that case the recall was lower (46.8%), values comparable with the Two-tiered binner of Saeed et al. (see Table 1). Three side populations were recovered as taxonomically consistent bins: the gamma proteobacterium related to *Thiotrichales* (5.3 Mb), already recovered by Saeed et al. (2011), a *Flavobacterium* (3.5 Mb), and a *Xanthomonas* (2.5 Mb).

The *Olavius algarvensis* symbiont metagenome (Woyke et al., 2006) consists of contigs of unknown origin and contigs belonging to three symbiotic bacteria: the Gamma-1, Gamma-3, and Delta-1 symbionts. The contigs of the Delta-1 and Gamma-3 symbionts were binned without problems (accuracy 90.6 and 98.1% respectively). The contigs of the Gamma-1 symbiont were difficult to separate from some contigs of unknown origin leading to a lower binning accuracy (76.5%) for this organism.

The contigs obtained from the Antarctic whale fall bone (Tringe et al., 2005) were not binned in the original study, so evaluation of the accuracy was impossible. However, as already reported by Saeed et al. (2011), they are binnable with modern methods. After tetranucleotide binning, two bins contained contigs of mainly Flavobacterial origin (the first bin was 3.9 Mb at 31.7% GC, the second 7.7 Mb at 39.7% GC). Four additional bins with a consistent taxonomic signature were recovered: a Pseudomonad bin (4.3 Mb, 45.5% GC), an Alteromonas bin (2.1 Mb at 40.5% GC), a *Rhodobacter* bin (3.2 Mb at 55.8% GC) and a Sphingomonas bin (3.8 Mb at 57.6% GC). The uncultured *Actinomycete* sequences previously reported were split over three different bins but were well separated from other organisms.

The acid mine drainage metagenome (Tyson et al., 2004) consists of contigs from five different populations: “*Ferroplasma acidarmanus* Type I”, “*Ferroplasma sp*. Type II”, “*Thermoplasmatales archaeon*,” “*Leptospirillum sp*. Group III,” and “*Leptospirillum sp*. Group II.” The contigs of the two *Ferroplasma’*s were binned together with some contamination of contigs from the *Thermoplasmatales archaeon*. The remainder of the *Thermoplasmatales archaeon* contigs were binned accurately in a separate bin. One bin contained only *Leptospirillum* Group III contigs and the final bin the *Leptospirillum* group II contigs with some contamination of *Leptospirillum* Group III contigs.

Once the benchmarking and testing was complete, the new binner was applied to eight metagenomes sampled from a microbial enrichment in a continuous culture mesocosm. The eight samples were tagged and sequenced on a single lane of an Illumina Genome Analyzer GA IIx instrument. Assembly yielded some reasonably long contigs and many short ones (Table 2), as usual in sequencing projects.

**Figure 3:**
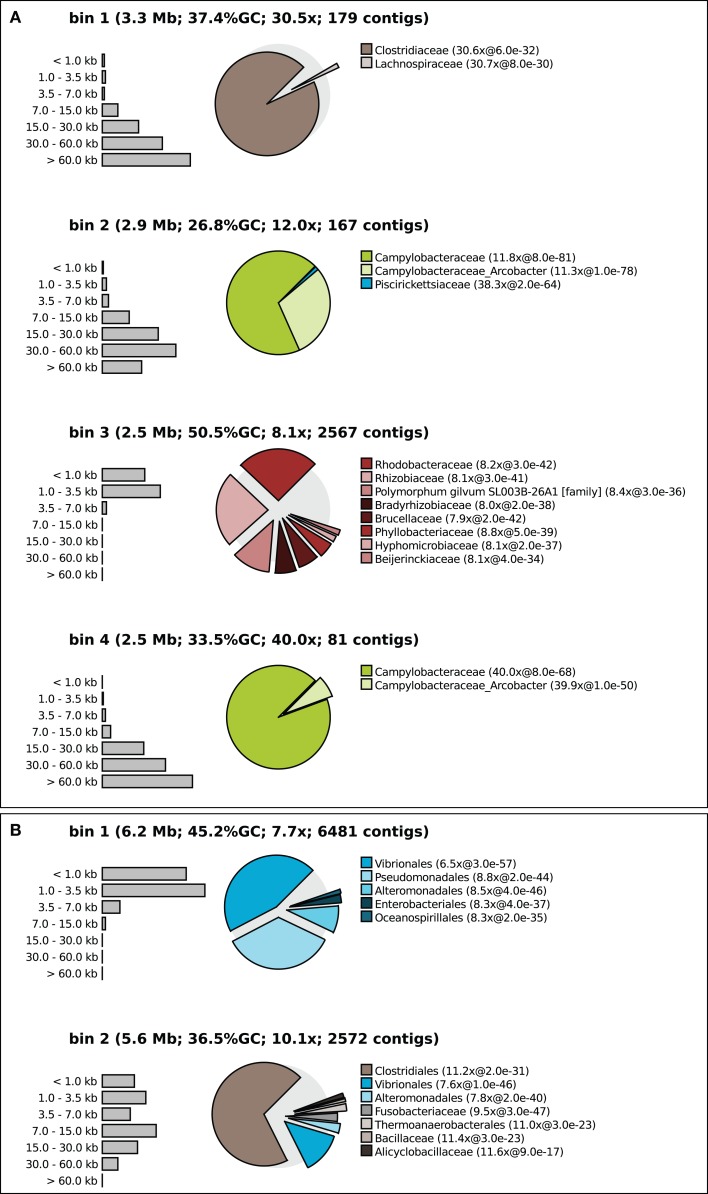
**Contig size distribution, sequencing coverage and taxonomic distribution of the four largest bins of sample 7 binned at medium confidence (A) and sample 1 binned at low confidence (B)**. The exploded pies show the taxonomic distribution of the bins. The distance of each part from the center of the pie is a measure for the median e-value of the associated hits (the larger the e-value the larger the distance from the center). Coverage is shown for the bin as a whole and separately for each pie part.

Tetranucleotide binning was performed as described above and again a taxonomic profile was calculated for each bin (Figure 3). The binner binned >99% of all contigs but the quality of the bins produced varied between samples, populations, and the applied threshold value. Figure 3A shows that the binning was apparently successful for sample 7, at medium threshold. Each of the bins had a distinct taxonomic signature as well as a distinct sequencing coverage. For example, apparently two different *Epsilonproteobacteria* (green colors) were present in this sample, one with a GC content of 26.8% and a sequencing coverage of 12 times and one with a slightly higher GC content and a coverage of 40 times. One of the bins may belong to an uncultured *Rhodobacter*, relatively unrelated to reference Alphaproteobacteria with sequenced genomes. This could be inferred from the relatively high BLAST *e*-values, and the scattering of the BLAST hits over different families. One may argue that this bin contains contigs of many different Alphaproteobacteria but this could be ruled out by inspecting other samples where this organism was more dominant and yielded longer contigs. The distribution of BLAST hits for individual contigs was very similar to the distribution of the bin as a whole. In fact, this is a nice example of what happens when supervised binning approaches (based on reference genomes) are applied to organisms only distantly related to those reference organisms – the contigs get scattered and are assigned to many different reference taxa.

Figure 3B shows an example of unsuccessful binning in sample 1 with a low confidence threshold (98% recall). Here, contigs from a *Pseudomonas* population appear to get mixed up with contigs from a *Vibrio* population in bin 1. Bin 2 contains sequences from *Vibrio* and a *Clostridum* populations. Note that these misbinnings were not observed at the high confidence threshold, but in that case the contigs of the *Vibrio* and the *Clostridium* were divided over many bins.

Interpolated Markov models yielded better results than tetranucleotide frequencies once sufficient training data was accumulated. Therefore, we again selected the good bins (long contigs, consistent taxonomic profile, as described above) from all samples to train IMM models. Eight distinct bins were identified that apparently defined the binnable part of the microbial community in all samples. All samples were binned *de novo* with these eight models and the resulting bins looked convincing in all cases, both with respect to coverage and taxonomic profile.

**Figure 4:**
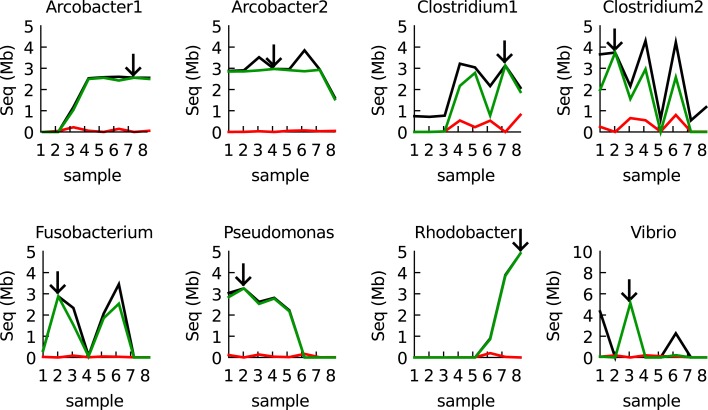
**Validation of the IMM binning results for the eight samples by BLAST**. The black line shows the total amount of assembled sequence information assigned to each of the eight populations by the IMM binner. The green line shows how much of that data was also recovered by BLAST (megaBLAST at 98% identity cutoff). The red line shows how much data was recovered in other bins (errors). Arrows indicate the origin of the reference bin used to train the IMM.

To further validate the results, BLAST was used to compare every sample with the eight reference bins (the bins used to construct the models, arrows in Figure 4). Only BLAST hits that were >98% identical to the reference sequence were considered. The IMM binning and the BLAST results were totally consistent, with two exceptions: (1) There was some cross binning of the two Clostridia populations. (2) The model predicted an abundant *Vibrio* population in sample 1 whereas this population could not be validated by BLAST. Apparently, the *Vibrio* population in sample 1 was different from the reference population in sample 3 (its contigs less than 98% identical to the contigs that constituted the defined *Vibrio* bins) and a different IMM model could be created for these contigs.

The community enriched in the continuous culture was provided with a marine medium with organic carbon as the only electron donor and nitrite as the electron acceptor. Organic carbon was present in excess, whereas nitrite was the limiting substrate. The binning results showed that the enriched community consisted of denitrifiers (affiliated with *Arcobacter*, *Pseudomonadales* or *Rhodobacterales*) and fermenters (affiliated with *Vibrionales, Clostridiales* and *Fusobacterales*). This is in agreement with textbook knowledge: one would expect the denitrifiers to consume most of the organic carbon while respiring nitrite, while the fermenters would consume the remainder of the carbon. Some dynamics appear to occur, but because of potential methodological biases between sequencing runs and assembly, this first needs to be confirmed with other methods (FISH). The biology of the experiment will be addressed in detail elsewhere once these data are avialable. The aim of the present study was to develop a method for metagenomic binning of these and future metagenomes sampled from laboratory enrichment cultures.

## Discussion

In this study we have shown that it is currently possible to bin metagenomic data obtained from relatively simple microbial communities with a modest sequencing effort. The eight samples investigated were tagged and sequenced on a single lane of an Illumina Genome analyzer GA IIx (consumable costs <5 kEuro). Our results also show that it is very important to sequence multiple samples from the same habitat: first, there appears to be quite some dynamics, even though these samples were obtained from the same bioreactor that was running at totally constant environmental conditions. Second, the possibility to assemble and bin a target population depends on the context of the overall community. Third the quality of the sequencing and/or assembly results differed between samples. This probably resulted from (unintended) differences during the manual library preparation and potentially a different degree of microdiversity among populations in different samples. Last but not least, by comparing results between samples with BLAST it was possible to validate the binning results and not depend on *a priori* estimates of binning accuracy.

The first step of the overall binning procedure makes use of a novel algorithm based on an empirical relationship between the mean and standard deviation of tetranucleotide frequencies. It has some advantages compared to existing methods: it does not depend on an estimate for the number of binnable populations, is open source, portable to any platform (that supports BLAST and Glimmer) and is extremely fast. The latter advantage also means that it is more scalable: it will be able to cope with the large amounts of data that will be produced by future sequencing technologies and can be applied to more complicated communities. By always running the binner at three different threshold values, all tested metagenomes could be binned successfully without the need for optimizing additional parameters.

This newly developed algorithm was combined with the use of IMM, already applied previously as a final “polishing” step in binning. Our study confirmed that IMMs outperform tetranucleotide frequencies when sufficient training data is available. However, where Kelley and Salzberg (2010) use a fully automated iterative method to refine the bins, we created the possibility for the scientist to choose which seed bins should be used to build the IMM models. This choice should be based on the characteristics of the bins such as contig length, sequencing coverage, and a taxonomic profile. We present evidence that this human intervention can outperform the fully automated method. This may be caused by difficulties in repairing totally failed bins (e.g., Figure 3B) by an iterative approach. In our enrichment culture metagenomes, iterations generally reallocated only a small amount of contigs (less than 50).

To facilitate the necessary human decisions, we implemented the complete procedure in Java Swing (the “Metawatt binner”) where the binning results are presented to the scientist as a graphical overview like the one displayed in Figure 3. This enables the selection of promising bins for IMM modeling. We made use of the Batik library to enable the export of these graphics in SVG format which can be directly used for publications. The produced bins can be exported as fasta files for further annotation in standard pipelines.

There is certainly room for improvement. Perhaps the biggest step forward could be achieved by integrating the assembly and the binning. Assembly speeds may increase when the assembler can be provided with compositional information, to more efficiently recruit promising reads for comparisons. “Associations” between contigs (with paired end reads) that are too weak to allow assembly directly may still be used for binning, as was recently shown by Iverson et al. (2012). Unfortunately the latter study provided no methodological details. Finally, next to sequencing coverage information, the frequency of single nucleotide polymorphisms may be used as an additional parameter to evaluate the binning results.

## Conclusion

We have developed and implemented a (partially) new approach for the binning of metagenomic contigs. This approach was born out of need, existing approaches did not produce satisfying results for our metagenomes. Evaluation of the binning accuracy and recall was done with artificial as well as real metagenomes and showed that it was comparable to the best existing approach tested. In addition several key improvements were realized. Most notably, the seeding of the bins does not depend on an estimate of the number of binnable populations and is very fast and scalable. The approach has been implemented in Java SWING as an open source application (the “Metawatt binner”) with an easy to use graphical user interface. Evaluation of the binning results by BLAST, training of models and manual editing of bins is included in the implementation.

Our results show that the metagenomic binning of relatively simple microbial communities is currently feasible even when the sequencing effort is moderate. We also show that it is important to sequence and compare metagenomes for multiple samples of the same habitat. Continuous culture of microbial enrichment combined with metagenomic sequencing is a powerful approach that can carry the study of microbial physiology from pure cultures to simple communities. An accurate and easy to use binning procedure is an essential aspect of this change.

## Author Contributions

Beate Kraft performed culturing and DNA extraction. Halina E. Tegetmeyer performed Illumina sequencing. Regina Bisdorf performed the assembly. Marc Strous developed and implemented the binning algorithm with help of Regina Bisdorf. The manuscript was written by Marc Strous with input from all other authors.

## Conflict of Interest Statement

The authors declare that the research was conducted in the absence of any commercial or financial relationships that could be construed as a potential conflict of interest.

## Supplementary Material

The Supplementary Material for this article can be found online at http://www.frontiersin.org/Microbial_Physiology_and_Metabolism/10.3389/fmicb.2012.00410/abstract

Supplementary Table S1 **Representative reference genomes used for deriving the empirical relationships shown in Figure 1**.

## Footnotes

^1^http://metavelvet.dna.bio.keio.ac.jp/

^2^http://xmlgraphics.apache.org/batik
