# Supplementary material for: The Binning of Metagenomic Contigs for Microbial Physiology of Mixed Cultures
Source: Front Microbiol. 2012 Dec 5;3:410. doi: 10.3389/fmicb.2012.00410 (PMC3514610; doi:10.3389/fmicb.2012.00410)
Supplement: Supplementary Table S1 — Representative reference genomes used for deriving the empirical relationships shown in Figure 1. [file 35527_Strous_DataSheet1.PDF]

Table S1: Representative reference genomes used for deriving the empirical relationships shown in Figure 1.

**Reference organism**	**Accession numbers**
Acaryochloris marina MBIC11017	NC_009925,NC_009926,NC_009927,NC_009928,NC_009929,NC_009930,NC_009931,NC_009932,NC_009933,NC_009934
Acetobacter pasteurianus IFO 3283-01	NC_013209,NC_013210,NC_013211,NC_013212,NC_013213,NC_013214,NC_013215
Acetohalobium arabaticum DSM 5501	NC_014378
Acholeplasma laidlawii PG-8A	NC_010163
Achromobacter xylosoxidans A8	NC_014640,NC_014641,NC_014642
Acidaminococcus fermentans DSM 20731	NC_013740
Acidianus hospitalis W1	NC_015518
Acidilobus saccharovorans 345-15	NC_014374
Acidimicrobium ferrooxidans DSM 10331	NC_013124
Acidiphilium cryptum JF-5	NC_009467,NC_009468,NC_009469,NC_009470,NC_009471,NC_009472,NC_009473,NC_009474,NC_009484
Acidithiobacillus caldus SM-1	NC_015850,NC_015851,NC_015852,NC_015853,NC_015854
Acidobacterium sp. MP5ACTX9	NC_015057,NC_015058,NC_015059,NC_015060,NC_015064,NC_015065
Acidobacterium capsulatum ATCC 51196	NC_012483
Acidothermus cellulolyticus 11B	NC_008578
Acidovorax sp. JS42	NC_008765,NC_008766,NC_008782
Aciduliprofundum boonei T469	NC_013926
Acinetobacter sp. ADP1	NC_005966
Actinobacillus pleuropneumoniae serovar 3 str. JL03	NC_010278

Reference organism	Accession numbers
Actinosynnema mirum DSM 43827	NC_013093
Aerococcus urinae ACS-120-V-Col10a	NC_015278
Aeromonas hydrophila subsp. hydrophila ATCC 7966	NC_008570
Aeropyrum pernix K1	NC_000854
Aggregatibacter actinomycetemcomitans D11S-1	NC_013416,NC_013438,NC_013597,NC_014629
Agrobacterium sp. H13-3	NC_015183,NC_015184,NC_015508
Akkermansia muciniphila ATCC BAA-835	NC_010655
Alcanivorax borkumensis SK2	NC_008260
Alicycliphilus denitrificans BC	NC_014908,NC_014910,NC_014911
Alicyclobacillus acidocaldarius subsp. acidocaldarius DSM 446	NC_013205,NC_013206,NC_013207,NC_013208
Aliivibrio salmonicida LFI1238	NC_011311,NC_011312,NC_011313,NC_011314,NC_011315,NC_011316
Alkalilimnicola ehrlichii MLHE-1	NC_008340
Alkaliphilus metalliredigens QYMF	NC_009633
Allochromatium vinosum DSM 180	NC_013851,NC_013852,NC_013862
Alteromonas sp. SN2	NC_015554
Aminobacterium colombiense DSM 12261	NC_014011
Ammonifex degensii KC4	NC_013385,NC_013386
Amycolatopsis mediterranei U32	NC_014318
Amycolicicoccus subflavus DQS3-9A1	NC_015560,NC_015561,NC_015564
Anabaena variabilis ATCC 29413	NC_007410,NC_007411,NC_007412,NC_007413,NC_014000
Anaerococcus prevotii DSM 20548	NC_013164,NC_013171
Anaerolinea thermophila UNI-1	NC_014960
Anaeromyxobacter sp. Fw109-5	NC_009675
Anaplasma centrale str. Israel	NC_013532

**Reference organism**	**Accession numbers**
Anoxybacillus flavithermus WK1	NC_011567
Aquifex aeolicus VF5	NC_000918,NC_001880
Arcanobacterium haemolyticum DSM 20595	NC_014218
Archaeoglobus fulgidus DSM 4304	NC_000917
Arcobacter butzleri RM4018	NC_009850
Aromatoleum aromaticum EbN1	NC_006513,NC_006823,NC_006824
Arthrobacter sp. FB24	NC_008537,NC_008538,NC_008539,NC_008541
Aster yellows witches'-broom phytoplasma AYWB	NC_007716,NC_007717,NC_007718,NC_007719,NC_007720
Asticcacaulis excentricus CB 48	NC_014816,NC_014817,NC_014818,NC_014819
Atopobium parvulum DSM 20469	NC_013203
Azoarcus sp. BH72	NC_008702
Azorhizobium caulinodans ORS 571	NC_009937
Azospirillum sp. B510	NC_013854,NC_013855,NC_013856,NC_013857,NC_013858,NC_013859,NC_013860
Azotobacter vinelandii DJ	NC_012560
Bacillus amyloliquefaciens DSM 7	NC_014551
Bacillus amyloliquefaciens FZB42	NC_009725
Bacillus anthracis str. A0248	NC_012655,NC_012656,NC_012659
Bacillus atrophaeus 1942	NC_014639
Bacillus cellulosilyticus DSM 2522	NC_014829
Bacillus clausii KSM-K16	NC_006582
Bacillus coagulans 2-6	NC_015634
Bacillus coagulans 36D1	NC_016023
Bacillus halodurans C-125	NC_002570

**Reference organism**	**Accession numbers**
Bacillus licheniformis ATCC 14580	NC_006270
Bacillus megaterium DSM 319	NC_014103
Bacillus megaterium QM B1551	NC_004604,NC_010008,NC_010009,NC_010010,NC_014019,NC_014023,NC_014025,NC_014031
Bacillus pseudofirmus OF4	NC_013791,NC_013792,NC_013793
Bacillus pumilus SAFR-032	NC_009848
Bacillus selenitireducens MLS10	NC_014219
Bacillus subtilis subsp. subtilis str. 168	NC_000964
Bacillus subtilis BSn5	NC_014976
Bacillus subtilis subsp. spizizenii TU-B-10	NC_016047
Bacillus subtilis subsp. spizizenii str. W23	NC_014479
Bacillus tusciae DSM 2912	NC_014098
Bacteroides fragilis NCTC 9343	NC_003228,NC_006873
Bartonella bacilliformis KC583	NC_008783
Baumannia cicadellinicola str. Hc (Homalodisca coagulata)	NC_007984
Bdellovibrio bacteriovorus HD100	NC_005363
Beijerinckia indica subsp. indica ATCC 9039	NC_010578,NC_010580,NC_010581
Beutenbergia cavernae DSM 12333	NC_012669
Bifidobacterium adolescentis ATCC 15703	NC_008618
Blattabacterium sp. (Blattella germanica) str. Bge	NC_013454,NC_015679
Bordetella avium 197N	NC_010645
Borrelia afzelii PKo	NC_008273,NC_008274,NC_008277,NC_008564,NC_008565,NC_008566,NC_008567,NC_008568,NC_008569,NT_167350,NT_167351,NT_167352

Reference organism	Accession numbers
Borrelia duttonii Ly	NC_011224,NC_011226,NC_011229,NC_011245,NC_011247,NC_011248,NC_011249,NC_011250,NC_011251,NC_011254,NC_011256,NC_011257,NC_011259,NC_011261,NC_011262,NC_011264,NC_011265
Borrelia hermsii DAH	NC_010673
Borrelia recurrentis A1	NC_011244,NC_011246,NC_011252,NC_011253,NC_011255,NC_011258,NC_011260,NC_011263
Borrelia turicatae 91E135	NC_008710
Brachybacterium faecium DSM 4810	NC_013172
Brachyspira hyodysenteriae WA1	NC_012225,NC_012226
Brachyspira murdochii DSM 12563	NC_014150
Brachyspira pilosicoli 95/1000	NC_014330
Bradyrhizobium sp. BTAi1	NC_009475,NC_009485
Brevibacillus brevis NBRC 100599	NC_012491
Brevundimonas subvibrioides ATCC 15264	NC_014375
Brucella abortus S19	NC_010740,NC_010742
Buchnera aphidicola str. 5A (Acyrthosiphon pisum)	NC_011833
Burkholderia sp. 383	NC_007509,NC_007510,NC_007511
Stenotrophomonas maltophilia JV3	NC_015947
Butyrivibrio proteoclasticus B316	NC_014387,NC_014388,NC_014389,NC_014390
Caldicellulosiruptor bescii DSM 6725	NC_012034,NC_012036,NC_012037
Calditerrivibrio nitroreducens DSM 19672	NC_014749,NC_014758
Caldivirga maquilingensis IC-167	NC_009954
Campylobacter concisus 13826	NC_009795,NC_009796,NC_009802

Reference organism	Accession numbers
Candidatus Accumulibacter phosphatis clade IIA str. UW-1	NC_013190,NC_013191,NC_013193,NC_013194
Candidatus Amoebophilus asiaticus 5a2	NC_010830
Candidatus Arthromitus sp. SFB-mouse-Japan	NC_015913
Candidatus Azobacteroides pseudotrichonymphae genomovar. CFP2	NC_011561,NC_011562,NC_011563,NC_011564,NC_011565
Candidatus Blochmannia floridanus	NC_005061
Candidatus Carsonella ruddii PV	NC_008512
Candidatus Chloracidobacterium thermophilum B	NC_016024,NC_016025
Candidatus Cloacamonas acidaminovorans	NS_000195
Candidatus Desulforudis audaxviator MP104C	NC_010424
Candidatus Hamiltonella defensa 5AT (Acyrthosiphon pisum)	NC_012751,NC_012752
Candidatus Hodgkinia cicadicola Dsem	NC_012960
Candidatus Korarchaeum cryptofilum OPF8	NC_010482
Candidatus Koribacter versatilis Ellin345	NC_008009
Candidatus Kuenenia stuttgartiensis	kusta,kustb,kustc,kustd,kuste
Candidatus Liberibacter asiaticus str. psy62	NC_012985
Candidatus Midichloria mitochondrii IricVA	NC_015722
Candidatus Moranella endobia PCIT	NC_015735
Candidatus Nitrospira defluvii	NC_014355
Candidatus Pelagibacter sp. IMCC9063	NC_015380
Candidatus Protochlamydia amoebophila UWE25	NC_005861
Candidatus Puniceispirillum marinum IMCC1322	NC_014010
Candidatus Riesia pediculicola USDA	NC_013962,NC_014109
Candidatus Ruthia magnifica str. Cm (Calyptogena magnifica)	NC_008610
Candidatus Solibacter usitatus Ellin6076	NC_008536

**Reference organism**	**Accession numbers**
Candidatus Sulcia muelleri CARI	NC_014499
Candidatus Sulcia muelleri DMIN	NC_014004
Candidatus Sulcia muelleri GWSS	NC_010118
Candidatus Sulcia muelleri SMDSEM	NC_013123
Candidatus Tremblaya princeps PCIT	NC_015736
Candidatus Vesicomyosocius okutanii HA	NC_009465
Candidatus Zinderia insecticola CARI	NC_014497
Capnocytophaga canimorsus Cc5	NC_015846
Carboxydothermus hydrogenoformans Z-2901	NC_007503
Carnobacterium sp. 17-4	NC_015390,NC_015391
Catenulispora acidiphila DSM 44928	NC_013131
Caulobacter sp. K31	NC_010333,NC_010335,NC_010338
Cellulomonas fimi ATCC 484	NC_015514
Cellulophaga algicola DSM 14237	NC_014934
Cellvibrio japonicus Ueda107	NC_010995
Cenarchaeum symbiosum A	NC_014820
Chelativorans sp. BNC1	NC_008242,NC_008243,NC_008244,NC_008254
Chitinophaga pinensis DSM 2588	NC_013132
Chlamydia muridarum Nigg	NC_002182,NC_002620
Chlamydophila abortus S26/3	NC_004552
Chlorobaculum parvum NCIB 8327	NC_011027
Chlorobium chlorochromatii CaD3	NC_007514
Chloroflexus sp. Y-400-fl	NC_012032
Chloroflexus aggregans DSM 9485	NC_011831
Chloroflexus aurantiacus J-10-fl	NC_010175
Chloroherpeton thalassium ATCC 35110	NC_011026
Chromobacterium violaceum ATCC 12472	NC_005085

**Reference organism**	**Accession numbers**
Chromohalobacter salexigens DSM 3043	NC_007963
Citrobacter koseri ATCC BAA-895	NC_009792,NC_009793,NC_009794
Clavibacter michiganensis subsp. michiganensis NCPPB 382	NC_009478,NC_009479,NC_009480
Clostridiales genomosp. BVAB3 str. UPII9-5	NC_013895
Clostridium sp. SY8519	NC_015737
Clostridium lentocellum DSM 5427	NC_015275
Collimonas fungivorans Ter331	NC_015856
Colwellia psychrerythraea 34H	NC_003910
Comamonas testosteroni CNB-2	NC_010935,NC_013446
Conexibacter woesei DSM 14684	NC_013739
Coprothermobacter proteolyticus DSM 5265	NC_011295
Coraliomargarita akajimensis DSM 45221	NC_014008
Corynebacterium aurimucosum ATCC 700975	NC_010813,NC_012590
Coxiella burnetii CbuG_Q212	NC_011527
Croceibacter atlanticus HTCC2559	NC_014230
Cronobacter sakazakii ATCC BAA-894	NC_009778,NC_009779,NC_009780
Cupriavidus metallidurans CH34	NC_007971,NC_007972,NC_007973,NC_007974
Cyanothece sp. ATCC 51142	NC_010539,NC_010541,NC_010542,NC_010543,NC_010546,NC_010547
Cyanothece sp. PCC 7424	NC_011729,NC_011730,NC_011732,NC_011733,NC_011734,NC_011737,NC_011738
Cyanothece sp. PCC 7425	NC_011880,NC_011882,NC_011884,NC_011885
Cyanothece sp. PCC 7822	NC_014501,NC_014502,NC_014503,NC_014504,NC_014533,NC_014534,NC_014

Reference organism	Accession numbers
	535
Cyanothece sp. PCC 8801	NC_011721,NC_011723,NC_011726,NC_011727
Cyanothece sp. PCC 8802	NC_013160,NC_013161,NC_013163,NC_013167,NC_013168
Cyclobacterium marinum DSM 745	NC_015914
Cytophaga hutchinsonii ATCC 33406	NC_008255
Dechloromonas aromatica RCB	NC_007298
Deferribacter desulfuricans SSM1	NC_013939,NC_013940
Dehalococcoides sp. BAV1	NC_009455
Dehalococcoides sp. CBDB1	NC_007356
Dehalococcoides sp. GT	NC_013890
Dehalococcoides sp. VS	NC_013552
Dehalococcoides ethenogenes 195	NC_002936
Dehalogenimonas lykanthroporepellens BL-DC-9	NC_014314
Deinococcus deserti VCD115	NC_012526,NC_012527,NC_012528,NC_012529
Delftia sp. Cs1-4	NC_015563
Denitrovibrio acetiphilus DSM 12809	NC_013943
Desulfarculus baarsii DSM 2075	NC_014365
Desulfatibacillum alkenivorans AK-01	NC_011768
Desulfitobacterium hafniense DCB-2	NC_011830
Desulfobacca acetoxidans DSM 11109	NC_015388
Desulfobacterium autotrophicum HRM2	NC_012108,NC_012109
Desulfobulbus propionicus DSM 2032	NC_014972
Desulfococcus oleovorans Hxd3	NC_009943
Desulfohalobium retbaense DSM 5692	NC_013223,NC_013224

Reference organism	Accession numbers
Desulfomicrobium baculatum DSM 4028	NC_013173
Desulfotalea psychrophila LSv54	NC_006138,NC_006139,NC_006140
Desulfotomaculum acetoxidans DSM 771	NC_013216
Desulfovibrio aespoeensis Aspo-2	NC_014844
Desulfurispirillum indicum S5	NC_014836
Desulfurivibrio alkaliphilus AHT2	NC_014216
Desulfurobacterium thermolithotrophum DSM 11699	NC_015185
Desulfurococcus kamchatkensis 1221n	NC_011766
Dichelobacter nodosus VCS1703A	NC_009446
Dickeya dadantii 3937	NC_014500
Dictyoglomus thermophilum H-6-12	NC_011297
Dictyoglomus turgidum DSM 6724	NC_011661
Dinoroseobacter shibae DFL 12	NC_009952,NC_009955,NC_009956,NC_009957,NC_009958,NC_009959
Dyadobacter fermentans DSM 18053	NC_013037
Edwardsiella ictaluri 93-146	NC_012779
Ehrlichia canis str. Jake	NC_007354
Elusimicrobium minutum Pei191	NC_010644
Enterobacter sp. 638	NC_009425,NC_009436
Enterococcus faecalis V583	NC_004668,NC_004669,NC_004670,NC_004671
Erwinia amylovora ATCC 49946	NC_013971,NC_013972,NC_013973
Erysipelothrix rhusiopathiae str. Fujisawa	NC_015601
Erythrobacter litoralis HTCC2594	NC_007722
Escherichia coli 536	NC_008253
Ethanoligenens harbinense YUAN-3	NC_014828
Eubacterium eligens ATCC 27750	NC_012778,NC_012780,NC_012782
Exiguobacterium sp. AT1b	NC_012673

Reference organism	Accession numbers
Exiguobacterium sibiricum 255-15	NC_010549,NC_010550,NC_010556
Ferrimonas balearica DSM 9799	NC_014541
Ferroglobus placidus DSM 10642	NC_013849
Fervidobacterium nodosum Rt17-B1	NC_009718
Fibrobacter succinogenes subsp. succinogenes S85	NC_013410
Finegoldia magna ATCC 29328	NC_010371,NC_010376
Flavobacteriaceae bacterium 3519-10	NC_013062
Maribacter sp. HTCC2170	NC_014472
Flavobacterium branchiophilum FL-15	NC_016001
Flexistipes sinusarabici DSM 4947	NC_015672
Fluviicola taffensis DSM 16823	NC_015321
Francisella sp. TX077308	NC_015696
Frankia sp. CcI3	NC_007777
Fusobacterium nucleatum subsp. nucleatum ATCC 25586	NC_003454
Gallibacterium anatis UMN179	NC_015460,NC_015461
Gallionella capsiferriformans ES-2	NC_014394
Gardnerella vaginalis 409-05	NC_013721
Gemmatimonas aurantiaca T-27	NC_012489
Geobacillus sp. C56-T3	NC_014206
Geobacillus sp. WCH70	NC_012790,NC_012793,NC_012794
Geobacillus sp. Y412MC52	NC_014915,NC_014916
Geobacillus sp. Y412MC61	NC_013411,NC_013412
Geobacillus sp. Y4.1MC1	NC_014650,NC_014651
Geobacillus kaustophilus HTA426	NC_006509,NC_006510
Geobacillus thermodenitrificans NG80-2	NC_009328,NC_009329
Geobacillus thermoglucosidasius C56-YS93	NC_015660,NC_015661,NC_015665
Geobacter sp. FRC-32	NC_011979
Geodermatophilus obscurus DSM 43160	NC_013757

**Reference organism**	**Accession numbers**
Glaciecola sp. 4H-3-7+YE-5	NC_015497,NC_015498
Gloeobacter violaceus PCC 7421	NC_005125
Gluconacetobacter diazotrophicus PAl 5	NC_011365,NC_011367
Gluconobacter oxydans 621H	NC_006672,NC_006673,NC_006674,NC_006675,NC_006676,NC_006677
Gordonia bronchialis DSM 43247	NC_013441,NC_013442
Gramella forsetii KT0803	NC_008571
Granulibacter bethesdensis CGDNIH1	NC_008343
Haemophilus ducreyi 35000HP	NC_002940
Haemophilus somnus 129PT	NC_006298,NC_008309
Hahella chejuensis KCTC 2396	NC_007645
Halalkalicoccus jeotgali B3	NC_014297,NC_014298,NC_014299,NC_014300,NC_014301,NC_014302,NC_014303
Halanaerobium hydrogeniformans	NC_014654
Haliangium ochraceum DSM 14365	NC_013440
Haliscomenobacter hydrossis DSM 1100	NC_015510,NC_015511,NC_015512,NC_015513
Haloarcula hispanica ATCC 33960	NC_015943,NC_015944,NC_015948
Halobacterium sp. NRC-1	NC_001869,NC_002607,NC_002608
Haloferax volcanii DS2	NC_013964,NC_013965,NC_013966,NC_013967,NC_013968
Halogeometricum borinquense DSM 11551	NC_014729,NC_014731,NC_014732,NC_014735,NC_014736,NC_014737
Halomicrobium mukohataei DSM 12286	NC_013201,NC_013202
Halomonas elongata DSM 2581	NC_014532
Halopiger xanaduensis SH-6	NC_015658,NC_015659,NC_015666,NC_015667

Reference organism	Accession numbers
Haloquadratum walsbyi DSM 16790	NC_008212,NC_008213
Halorhabdus utahensis DSM 12940	NC_013158
Halorhodospira halophila SL1	NC_008789
Halorubrum lacusprofundi ATCC 49239	NC_012028,NC_012029,NC_012030
Haloterrigena turkmenica DSM 5511	NC_013743,NC_013744,NC_013745,NC_013746,NC_013747,NC_013748,NC_013749
Halothermothrix orenii H 168	NC_011899
Halothiobacillus neapolitanus c2	NC_013422
Helicobacter acinonychis str. Sheeba	NC_008229,NC_008230
Heliobacterium modesticaldum Ice1	NC_010337
Herbaspirillum seropedicae SmR1	NC_014323
Herminiimonas arsenicoxydans	NC_009138
Herpetosiphon aurantiacus DSM 785	NC_009972,NC_009973,NC_009974
Hippea maritima DSM 10411	NC_015318
Hirschia baltica ATCC 49814	NC_012982,NC_012983
Hydrogenobacter thermophilus TK-6	NC_013799
Hydrogenobaculum sp. Y04AAS1	NC_011126
Hyperthermus butylicus DSM 5456	NC_008818
Hyphomicrobium sp. MC1	NC_015717
Hyphomonas neptunium ATCC 15444	NC_008358
Idiomarina loihiensis L2TR	NC_006512
Ignicoccus hospitalis KIN4/I	NC_009776
Ignisphaera aggregans DSM 17230	NC_014471
Ilyobacter polytropus DSM 2926	NC_014632,NC_014633,NC_014634
Intrasporangium calvum DSM 43043	NC_014830
Isoptericola variabilis 225	NC_015588
Isosphaera pallida ATCC 43644	NC_014957,NC_014962

**Reference organism**	**Accession numbers**
Jannaschia sp. CCS1	NC_007801,NC_007802
Janthinobacterium sp. Marseille	NC_009659
Jonesia denitrificans DSM 20603	NC_013174
Kangiella koreensis DSM 16069	NC_013166
Ketogulonicigenium vulgare Y25	NC_014621,NC_014625,NC_014626
Kineococcus radiotolerans SRS30216	NC_009660,NC_009664,NC_009806
Kitasatospora setae KM-6054	NC_016109
Klebsiella pneumoniae 342	NC_011281,NC_011282,NC_011283
Kosmotoga olearia TBF 19.5.1	NC_012785
Kribbella flavida DSM 17836	NC_013729
Krokinobacter sp. 4H-3-7-5	NC_015496
Kytococcus sedentarius DSM 20547	NC_013169
Lacinutrix sp. 5H-3-7-4	NC_015638
Lactobacillus acidophilus 30SC	NC_015213,NC_015214,NC_015218
Lactobacillus acidophilus NCFM	NC_006814
Lactobacillus amylovorus GRL 1112	NC_014724,NC_015319,NC_015322
Lactobacillus brevis ATCC 367	NC_008497,NC_008498,NC_008499
Lactobacillus buchneri NRRL B-30929	NC_015420,NC_015421,NC_015428,NC_015429
Lactobacillus casei ATCC 334	NC_008502,NC_008526
Lactobacillus casei BL23	NC_010999
Lactobacillus casei str. Zhang	NC_011352,NC_014334
Lactobacillus crispatus ST1	NC_014106
Lactobacillus delbrueckii subsp. bulgaricus ATCC 11842	NC_008054
Lactobacillus delbrueckii subsp. bulgaricus ATCC BAA-365	NC_008529
Lactobacillus delbrueckii subsp. bulgaricus ND02	NC_014727,NC_014728
Lactobacillus fermentum IFO 3956	NC_010610

Reference organism	Accession numbers
Lactobacillus gasseri ATCC 33323	NC_008530
Lactobacillus helveticus DPC 4571	NC_010080
Lactobacillus johnsonii FI9785	NC_012552,NC_013504,NC_013505
Lactobacillus johnsonii NCC 533	NC_005362
Lactobacillus kefiranofaciens ZW3	NC_015598,NC_015602,NC_015603
Lactobacillus plantarum JDM1	NC_012984
Lactobacillus plantarum subsp. plantarum ST-III	NC_014554,NC_014558
Lactobacillus plantarum WCFS1	NC_004567,NC_006375,NC_006376,NC_006377
Lactobacillus reuteri DSM 20016	NC_009513
Lactobacillus reuteri JCM 1112	NC_010609
Lactobacillus reuteri SD2112	NC_015697,NC_015698,NC_015699,NC_015700,NC_015701
Lactobacillus rhamnosus GG	NC_013198
Lactobacillus rhamnosus Lc 705	NC_013199,NC_013200
Lactobacillus ruminis ATCC 27782	NC_015975
Lactobacillus sakei subsp. sakei 23K	NC_007576
Lactobacillus salivarius UCC118	NC_006529,NC_006530,NC_007929,NC_007930
Lactobacillus sanfranciscensis TMW 1.1304	NC_015978,NC_015979,NC_015980
Lactococcus garvieae ATCC 49156	NC_015930
Lactococcus lactis subsp. lactis Il1403	NC_002662
Lactococcus lactis subsp. lactis KF147	NC_013656,NC_013657
Lactococcus lactis subsp. cremoris MG1363	NC_009004
Lactococcus lactis subsp. cremoris SK11	NC_008503,NC_008504,NC_008505,NC_008506,NC_008507,NC_008527
Laribacter hongkongensis HLHK9	NC_012559
Lawsonia intracellularis PHE/MN1-00	NC_008011,NC_008012,NC_008013,NC_

Reference organism	Accession numbers
	008014
Leadbetterella byssophila DSM 17132	NC_014655
Legionella longbeachae NSW150	NC_013861,NC_014544
Leptospira biflexa serovar Patoc strain 'Patoc 1 (Ames)'	NC_010842,NC_010845,NC_010846
Leptospira biflexa serovar Patoc strain 'Patoc 1 (Paris)'	NC_010602,NC_010843,NC_010844
Leptospira borgpetersenii serovar Hardjo-bovis JB197	NC_008510,NC_008511
Leptospira borgpetersenii serovar Hardjo-bovis L550	NC_008508,NC_008509
Leptospira interrogans serovar Copenhageni str. Fiocruz L1-130	NC_005823,NC_005824
Leptospira interrogans serovar Lai str. 56601	NC_004342,NC_004343
Leptothrix cholodnii SP-6	NC_010524
Leptotrichia buccalis C-1013-b	NC_013192
Leuconostoc sp. C2	NC_015734
Leuconostoc citreum KM20	NC_010466,NC_010467,NC_010469,NC_010470,NC_010471
Leuconostoc gasicomitatum LMG 18811	NC_014319
Leuconostoc kimchii IMSNU 11154	NC_014131,NC_014132,NC_014133,NC_014134,NC_014135,NC_014136
Leuconostoc mesenteroides subsp. mesenteroides ATCC 8293	NC_008496,NC_008531
Listeria innocua Clip11262	NC_003212,NC_003383
Listeria ivanovii subsp. ivanovii PAM 55	NC_016011
Listeria monocytogenes 08-5923	NC_013768
Listeria monocytogenes serotype 4b str. CLIP 80459	NC_012488
Listeria monocytogenes EGD-e	NC_003210
Listeria monocytogenes HCC23	NC_011660
Listeria monocytogenes serotype 4b str. F2365	NC_002973
Listeria monocytogenes 08-5578	NC_013766,NC_013767

Reference organism	Accession numbers
Listeria seeligeri serovar 1/2b str. SLCC3954	NC_013891
Listeria welshimeri serovar 6b str. SLCC5334	NC_008555
Lysinibacillus sphaericus C3-41	NC_010381,NC_010382
Macrococcus caseolyticus JCSC5402	NC_011995,NC_011996,NC_011997,NC_011998,NC_011999,NC_012000,NC_012001,NC_012002,NC_012003
Magnetococcus sp. MC-1	NC_008576
Magnetospirillum magneticum AMB-1	NC_007626
Mahella australiensis 50-1 BON	NC_015520
Mannheimia succiniciproducens MBEL55E	NC_006300
Maricaulis maris MCS10	NC_008347
Marinithermus hydrothermalis DSM 14884	NC_015387
Marinobacter aquaeolei VT8	NC_008738,NC_008739,NC_008740
Marinomonas sp. MWYL1	NC_009654
Marinomonas mediterranea MMB-1	NC_015276
Marinomonas posidonica IVIA-Po-181	NC_015559
Marivirga tractuosa DSM 4126	NC_014750,NC_014759
Megasphaera elsdenii DSM 20460	NC_015873
Meiothermus ruber DSM 1279	NC_013946
Melissococcus plutonius ATCC 35311	NC_015516,NC_015517
Mesoplasma florum L1	NC_006055
Mesorhizobium ciceri biovar biserrulae WSM1271	NC_014918,NC_014923
Metallosphaera cuprina Ar-4	NC_015435
Methanobacterium sp. AL-21	NC_015216
Methanobrevibacter ruminantium M1	NC_013790
Methanocaldococcus sp. FS406-22	NC_013887,NC_013888
Methanocella paludicola SANAE	NC_013665
Methanococcoides burtonii DSM 6242	NC_007955

**Reference organism**	**Accession numbers**
Methanococcus aeolicus Nankai-3	NC_009635
Methanocorpusculum labreanum Z	NC_008942
Methanoculleus marisnigri JR1	NC_009051
Methanohalobium evestigatum Z-7303	NC_014253,NC_014254
Methanohalophilus mahii DSM 5219	NC_014002
Methanoplanus petrolearius DSM 11571	NC_014507
Methanopyrus kandleri AV19	NC_003551
Methanoregula boonei 6A8	NC_009712
Methanosaeta concilii GP6	NC_015416,NC_015430
Methanosalsum zhilinae DSM 4017	NC_015676
Methanosarcina acetivorans C2A	NC_003552
Methanosphaera stadtmanae DSM 3091	NC_007681
Methanosphaerula palustris E1-9c	NC_011832
Methanospirillum hungatei JF-1	NC_007796
Methanothermobacter marburgensis str. Marburg	NC_014408,NC_014409
Methanothermococcus okinawensis IH1	NC_015632,NC_015636
Methanothermus fervidus DSM 2088	NC_014658
Methanotorris igneus Kol 5	NC_015562
Methylacidiphilum infernorum V4	NC_010794
Methylibium petroleiphilum PM1	NC_008825,NC_008826
Methylobacillus flagellatus KT	NC_007947
Methylobacterium sp. 4-46	NC_010373,NC_010374,NC_010511
Methylocella silvestris BL2	NC_011666
Methylococcus capsulatus str. Bath	NC_002977
Methylomicrobium alcaliphilum	NC_016112
Methylomirabilis oxyfera	damo
Methylomonas methanica MC09	NC_015572
Methylotenera versatilis 301	NC_014207

**Reference organism**	**Accession numbers**
Methylovorus sp. MP688	NC_014733
Micavibrio aeruginosavorus ARL-13	NC_016026
Microcystis aeruginosa NIES-843	NC_010296
Microlunatus phosphovorus NM-1	NC_015635
Micromonospora sp. L5	NC_014815
Moraxella catarrhalis RH4	NC_014147
Muricauda ruestringensis DSM 13258	NC_015945
Mycoplasma agalactiae PG2	NC_009497
Mycoplasma agalactiae	NC_013948
Mycoplasma arthritidis 158L3-1	NC_011025
Mycoplasma bovis Hubei-1	NC_015725
Mycoplasma bovis PG45	NC_014760
Mycoplasma capricolum subsp. capricolum ATCC 27343	NC_007633
Mycoplasma conjunctivae HRC/581	NC_012806
Mycoplasma crocodyli MP145	NC_014014
Mycoplasma fermentans JER	NC_014552
Mycoplasma fermentans M64	NC_014921
Mycoplasma gallisepticum str. R(low)	NC_004829
Mycoplasma genitalium G37	NC_000908
Mycoplasma haemofelis str. Langford 1	NC_014970
Mycoplasma hominis ATCC 23114	NC_013511
Mycoplasma hyopneumoniae 232	NC_006360
Mycoplasma hyopneumoniae 7448	NC_007332
Mycoplasma hyopneumoniae J	NC_007295
Mycoplasma hyorhinis HUB-1	NC_014448
Mycoplasma leachii PG50	NC_014751
Mycoplasma mobile 163K	NC_006908
Mycoplasma mycoides subsp. mycoides SC str. PG1	NC_005364

Reference organism	Accession numbers
Mycoplasma mycoides subsp. capri LC str. 95010	NC_015407,NC_015431
Mycoplasma penetrans HF-2	NC_004432
Mycoplasma pneumoniae M129	NC_000912
Mycoplasma pulmonis UAB CTIP	NC_002771
Mycoplasma putrefaciens KS1	NC_015946
Mycoplasma suis str. Illinois	NC_015155
Mycoplasma suis KI3806	NC_015153
Mycoplasma synoviae 53	NC_007294
Nakamurella multipartita DSM 44233	NC_013235
Nanoarchaeum equitans Kin4-M	NC_005213
Natranaerobius thermophilus JW/NM-WN-LF	NC_010715,NC_010718,NC_010724
Natrialba magadii ATCC 43099	NC_013922,NC_013923,NC_013924,NC_013925
Natronomonas pharaonis DSM 2160	NC_007426,NC_007427,NC_007428
Nautilia profundicola AmH	NC_012115
Neisseria gonorrhoeae FA 1090	NC_002946
Neorickettsia risticii str. Illinois	NC_013009
Nitratifractor salsuginis DSM 16511	NC_014935
Nitratiruptor sp. SB155-2	NC_009662
Nitrobacter hamburgensis X14	NC_007959,NC_007960,NC_007961,NC_007964
Nitrosococcus halophilus Nc4	NC_013958,NC_013960
Nitrosomonas sp. AL212	NC_015221,NC_015222,NC_015223
Nitrosopumilus maritimus SCM1	NC_010085
Nitrosospira multiformis ATCC 25196	NC_007614,NC_007615,NC_007616,NC_007617
Nocardia farcinica IFM 10152	NC_006361,NC_006362,NC_006363
Nocardiopsis dassonvillei subsp. dassonvillei DSM 43111	NC_014210,NC_014211
Nostoc sp. PCC 7120	NC_003240,NC_003241,NC_003267,NC_003270,NC_003272,NC_003273,NC_003276
Nostoc punctiforme PCC 73102	NC_010628,NC_010629,NC_010630,NC_010631,NC_010632,NC_010633
Novosphingobium sp. PP1Y	NC_015579,NC_015580,NC_015582,NC_015583
Oceanithermus profundus DSM 14977	NC_014753,NC_014761
Oceanobacillus iheyensis HTE831	NC_004193
Ochrobactrum anthropi ATCC 49188	NC_009667,NC_009668,NC_009669,NC_009670,NC_009671,NC_009672
Odoribacter splanchnicus DSM 20712	NC_015160
Oenococcus oeni PSU-1	NC_008528

Reference organism	Accession numbers
Oligotropha carboxidovorans OM5	NC_011386
Opitutus terrae PB90-1	NC_010571
Orientia tsutsugamushi str. Boryong	NC_009488
Oscillibacter valericigenes Sjm18-20	NC_016046,NC_016048
Paenibacillus sp. JDR-2	NC_012914
Paenibacillus sp. Y412MC10	NC_013406
Paenibacillus mucilaginosus KNP414	NC_015690
Paenibacillus polymyxa E681	NC_014483
Paenibacillus polymyxa SC2	NC_014622,NC_014628
Paludibacter propionicigenes WB4	NC_014734
Pantoea sp. At-9b	NC_014837,NC_014838,NC_014839,NC_014840,NC_014841,NC_014842
Parabacteroides distasonis ATCC 8503	NC_009615
Parachlamydia acanthamoebae UV7	NC_015702
Paracoccus denitrificans PD1222	NC_008686,NC_008687,NC_008688
Parvibaculum lavamentivorans DS-1	NC_009719
Parvularcula bermudensis HTCC2503	NC_014414
Pasteurella multocida subsp. multocida str. Pm70	NC_002663
Pectobacterium atrosepticum SCRI1043	NC_004547
Pediococcus pentosaceus ATCC 25745	NC_008525
Pedobacter heparinus DSM 2366	NC_013061
Pelagibacterium halotolerans B2	NC_016078,NC_016079
Pelobacter carbinolicus DSM 2380	NC_007498
Pelotomaculum thermopropionicum SI	NC_009454
Persephonella marina EX-H1	NC_012439,NC_012440
Petrotoga mobilis SJ95	NC_010003
Phenylobacterium zucineum HLK1	NC_011143,NC_011144
Photobacterium profundum SS9	NC_005871,NC_006370,NC_006371
Photorhabdus asymbiotica subsp. asymbiotica ATCC 43949	NC_012961,NC_012962
Picrophilus torridus DSM 9790	NC_005877
Pirellula staleyi DSM 6068	NC_013720
Planctomyces brasiliensis DSM 5305	NC_015174
Polaromonas sp. JS666	NC_007948,NC_007949,NC_007950
Polymorphum gilvum SL003B-26A1	NC_015258,NC_015259
Polynucleobacter necessarius subsp. necessarius STIR1	NC_010531
Porphyromonas asaccharolytica DSM 20707	NC_015501
Prevotella denticola F0289	NC_015311
Prochlorococcus marinus str. AS9601	NC_008816
Prochlorococcus marinus subsp. marinus str. CCMP1375	NC_005042
Prochlorococcus marinus str. MIT 9211	NC_009976

Reference organism	Accession numbers
Prochlorococcus marinus str. MIT 9215	NC_009840
Prochlorococcus marinus str. MIT 9301	NC_009091
Prochlorococcus marinus str. MIT 9303	NC_008820
Prochlorococcus marinus str. MIT 9312	NC_007577
Prochlorococcus marinus str. MIT 9313	NC_005071
Prochlorococcus marinus str. MIT 9515	NC_008817
Prochlorococcus marinus str. NATL1A	NC_008819
Prochlorococcus marinus str. NATL2A	NC_007335
Prochlorococcus marinus subsp. pastoris str. CCMP1986	NC_005072
Prosthecochloris aestuarii DSM 271	NC_011059,NC_011061
Proteus mirabilis HI4320	NC_010554,NC_010555
Pseudoalteromonas sp. SM9913	NC_014800,NC_014803
Pseudogulbenkiania sp. NH8B	NC_016002
Pseudomonas aeruginosa LESB58	NC_011770
Pseudoxanthomonas spadix BD-a59	NC_016147
Psychrobacter sp. PRwf-1	NC_009516,NC_009517,NC_009524
Psychromonas ingrahamii 37	NC_008709
Pusillimonas sp. T7-7	NC_015458,NC_015459
Pyrobaculum aerophilum str. IM2	NC_003364
Pyrococcus sp. NA2	NC_015474
Pyrolobus fumarii 1A	NC_015931
Rahnella sp. Y9602	NC_015061,NC_015062,NC_015063
Ralstonia pickettii 12D	NC_012849,NC_012851,NC_012855,NC_012856,NC_012857
Ramlibacter tataouinensis TTB310	NC_015677
Sinorhizobium fredii NGR234	NC_000914,NC_012586,NC_012587
Rhodobacter capsulatus SB 1003	NC_014034,NC_014035
Rhodoferax ferrireducens T118	NC_007901,NC_007908
Rhodomicrobium vannielii ATCC 17100	NC_014664
Rhodopirellula baltica SH 1	NC_005027
Rhodopseudomonas palustris BisA53	NC_008435
Rhodospirillum centenum SW	NC_011420
Rhodothermus marinus DSM 4252	NC_013501,NC_013502
Rhodothermus marinus SG0.5JP17-172	NC_015966,NC_015967,NC_015970
Riemerella anatipestifer DSM 15868	NC_014738
Robiginitalea biformata HTCC2501	NC_013222
Roseburia hominis A2-183	NC_015977
Roseiflexus sp. RS-1	NC_009523
Roseiflexus castenholzii DSM 13941	NC_009767
Roseobacter denitrificans OCh 114	NC_008209,NC_008386,NC_008387,NC_008388,NC_008389

Reference organism	Accession numbers
Rubrobacter xylanophilus DSM 9941	NC_008148
Ruegeria sp. TM1040	NC_008042,NC_008043,NC_008044
Ruminococcus albus 7	NC_014824,NC_014825,NC_014826,NC_014827,NC_014833
Runella slithyformis DSM 19594	NC_015693,NC_015694,NC_015695,NC_015703,NC_015704,NC_015705
Saccharophagus degradans 2-40	NC_007912
Salinibacter ruber DSM 13855	NC_007677,NC_007678
Salinibacter ruber M8	NC_014026,NC_014028,NC_014030,NC_014032
Salmonella bongori NCTC 12419	NC_015761
Sanguibacter keddieii DSM 10542	NC_013521
Sebaldella termitidis ATCC 33386	NC_013517,NC_013518,NC_013519
Segniliparus rotundus DSM 44985	NC_014168
Serratia sp. AS12	NC_015566
Shewanella sp. ANA-3	NC_008573,NC_008577
Shigella boydii CDC 3083-94	NC_010656,NC_010657,NC_010658,NC_010659,NC_010660,NC_010672
Sideroxydans lithotrophicus ES-1	NC_013959
Simkania negevensis Z	NC_015710,NC_015713
Sodalis glossinidius str. 'morsitans'	NC_007712,NC_007713,NC_007714,NC_007715
Sorangium cellulosum 'So ce 56'	NC_010162
Sphaerobacter thermophilus DSM 20745	NC_013523,NC_013524
Sphingobacterium sp. 21	NC_015277
Sphingobium sp. SYK-6	NC_015974,NC_015976
Sphingomonas wittichii RW1	NC_009507,NC_009508,NC_009511
Sphingopyxis alaskensis RB2256	NC_008036,NC_008048
Spirochaeta sp. Buddy	NC_015152
Spirochaeta caldaria DSM 7334	NC_015732
Spirochaeta coccoides DSM 17374	NC_015436
Spirochaeta smaragdinae DSM 11293	NC_014364
Spirochaeta thermophila DSM 6192	NC_014484
Spirosoma linguale DSM 74	NC_013730,NC_013731,NC_013732,NC_013733,NC_013734,NC_013735,NC_013736,NC_013737,NC_013738
Stackebrandtia nassauensis DSM 44728	NC_013947
Staphylococcus aureus subsp. aureus COL	NC_002951,NC_006629
Staphylococcus aureus subsp. aureus ED98	NC_013450,NC_013451,NC_013452,NC_013453
Staphylococcus aureus subsp. aureus JH1	NC_009619,NC_009632

Reference organism	Accession numbers
Staphylococcus aureus subsp. aureus JH9	NC_009477,NC_009487
Staphylococcus aureus subsp. aureus MRSA252	NC_002952
Staphylococcus aureus subsp. aureus MSSA476	NC_002953,NC_005951
Staphylococcus aureus subsp. aureus MW2	NC_003923
Staphylococcus aureus subsp. aureus Mu3	NC_009782
Staphylococcus aureus subsp. aureus Mu50	NC_002758,NC_002774
Staphylococcus aureus subsp. aureus N315	NC_002745,NC_003140
Staphylococcus aureus subsp. aureus NCTC 8325	NC_007795
Staphylococcus aureus subsp. aureus str. Newman	NC_009641
Staphylococcus aureus RF122	NC_007622
Staphylococcus aureus subsp. aureus USA300_FPR3757	NC_007790,NC_007791,NC_007792,NC_007793
Staphylococcus aureus subsp. aureus USA300_TCH1516	NC_010063,NC_010079,NC_012417
Staphylococcus carnosus subsp. carnosus TM300	NC_012121
Staphylococcus epidermidis ATCC 12228	NC_004461,NC_005003,NC_005004,NC_005005,NC_005006,NC_005007,NC_005008
Staphylococcus epidermidis RP62A	NC_002976,NC_006663
Staphylococcus haemolyticus JCSC1435	NC_007168,NC_007169,NC_007170,NC_007171
Staphylococcus lugdunensis HKU09-01	NC_013893
Staphylococcus pseudintermedius HKU10-03	NC_014925
Staphylococcus saprophyticus subsp. saprophyticus ATCC 15305	NC_007350,NC_007351,NC_007352
Staphylothermus hellenicus DSM 12710	NC_014205
Starkeya novella DSM 506	NC_014217
Streptobacillus moniliformis DSM 12112	NC_013515,NC_013516
Streptococcus agalactiae 2603V/R	NC_004116
Streptococcus agalactiae A909	NC_007432
Streptococcus agalactiae NEM316	NC_004368
Streptococcus dysgalactiae subsp. equisimilis GGS_124	NC_012891
Streptococcus equi subsp. equi 4047	NC_012471
Streptococcus equi subsp. zooepidemicus MGCS10565	NC_011134
Streptococcus equi subsp. zooepidemicus	NC_012470
Streptococcus gallolyticus subsp. gallolyticus ATCC BAA-2069	NC_015215,NC_015219
Streptococcus gallolyticus UCN34	NC_013798
Streptococcus gordonii str. Challis substr. CH1	NC_009785
Streptococcus mitis B6	NC_013853
Streptococcus mutans NN2025	NC_013928
Streptococcus mutans UA159	NC_004350

Reference organism	Accession numbers
Streptococcus oralis Uo5	NC_015291
Streptococcus parasanguinis ATCC 15912	NC_015678
Streptococcus parauberis KCTC 11537	NC_015558
Streptococcus pasteurianus ATCC 43144	NC_015600
Streptococcus pneumoniae 670-6B	NC_014498
Streptococcus pneumoniae 70585	NC_012468
Streptococcus pneumoniae AP200	NC_014494
Streptococcus pneumoniae ATCC 700669	NC_011900
Streptococcus pneumoniae CGSP14	NC_010582
Streptococcus pneumoniae D39	NC_008533
Streptococcus pneumoniae G54	NC_011072
Streptococcus pneumoniae Hungary19A-6	NC_010380
Streptococcus pneumoniae JJA	NC_012466
Streptococcus pneumoniae P1031	NC_012467
Streptococcus pneumoniae R6	NC_003098
Streptococcus pneumoniae TCH8431/19A	NC_014251
Streptococcus pneumoniae TIGR4	NC_003028
Streptococcus pneumoniae Taiwan19F-14	NC_012469
Streptococcus pseudopneumoniae IS7493	NC_015875,NC_015876
Streptococcus pyogenes M1 GAS	NC_002737
Streptococcus pyogenes MGAS10270	NC_008022
Streptococcus pyogenes MGAS10394	NC_006086
Streptococcus pyogenes MGAS10750	NC_008024
Streptococcus pyogenes MGAS2096	NC_008023
Streptococcus pyogenes MGAS315	NC_004070
Streptococcus pyogenes MGAS5005	NC_007297
Streptococcus pyogenes MGAS6180	NC_007296
Streptococcus pyogenes MGAS8232	NC_003485
Streptococcus pyogenes MGAS9429	NC_008021
Streptococcus pyogenes str. Manfredo	NC_009332
Streptococcus pyogenes NZ131	NC_011375
Streptococcus pyogenes SSI-1	NC_004606
Streptococcus salivarius CCHSS3	NC_015760
Streptococcus sanguinis SK36	NC_009009
Streptococcus suis 05ZYH33	NC_009442
Streptococcus suis 98HAH33	NC_009443
Streptococcus suis BM407	NC_012923,NC_012926
Streptococcus suis P1/7	NC_012925
Streptococcus suis SC84	NC_012924
Streptococcus suis ST3	NC_015433

Reference organism	Accession numbers
Streptococcus thermophilus CNRZ1066	NC_006449
Streptococcus thermophilus LMD-9	NC_008500,NC_008501,NC_008532
Streptococcus thermophilus LMG 18311	NC_006448
Streptococcus uberis 0140J	NC_012004
Streptosporangium roseum DSM 43021	NC_013595,NC_013596
Sulfobacillus acidophilus TPY	NC_015757
Sulfolobus acidocaldarius DSM 639	NC_007181
Sulfuricurvum kujiense DSM 16994	NC_014754,NC_014755,NC_014756,NC_014762,NC_014763
Sulfurihydrogenibium sp. YO3AOP1	NC_010730
Sulfurihydrogenibium azorense Az-Fu1	NC_012438
Sulfurimonas autotrophica DSM 16294	NC_014506
Sulfurospirillum deleyianum DSM 6946	NC_013512
Sulfurovum sp. NBC37-1	NC_009663
Symbiobacterium thermophilum IAM 14863	NC_006177
Synechococcus sp. CC9311	NC_008319
Synechococcus sp. CC9605	NC_007516
Synechococcus sp. CC9902	NC_007513
Synechococcus sp. JA-2-3B'a(2-13)	NC_007776
Synechococcus sp. JA-3-3Ab	NC_007775
Synechococcus sp. PCC 7002	NC_010474,NC_010475,NC_010476,NC_010477,NC_010478,NC_010479,NC_010480
Synechococcus sp. RCC307	NC_009482
Synechococcus sp. WH 7803	NC_009481
Synechococcus sp. WH 8102	NC_005070
Synechococcus elongatus PCC 6301	NC_006576
Synechococcus elongatus PCC 7942	NC_007595,NC_007604
Synechocystis sp. PCC 6803	NC_000911,NC_005229,NC_005230,NC_005231,NC_005232
Syntrophobacter fumaroxidans MPOB	NC_008554
Syntrophobotulus glycolicus DSM 8271	NC_015172
Syntrophomonas wolfei subsp. wolfei str. Goettingen	NC_008346
Syntrophothermus lipocalidus DSM 12680	NC_014220
Syntrophus aciditrophicus SB	NC_007759
Taylorella asinigenitalis MCE3	NC_016043
Tepidanaerobacter sp. Re1	NC_015519
Teredinibacter turnerae T7901	NC_012997
Terriglobus saanensis SP1PR4	NC_014963
Tetragenococcus halophilus NBRC 12172	NC_016052
Thauera sp. MZ1T	NC_011662,NC_011667

Reference organism	Accession numbers
Thermaerobacter marianensis DSM 12885	NC_014831
Thermanaerovibrio acidaminovorans DSM 6589	NC_013522
Thermincola potens JR	NC_014152
Thermoanaerobacter sp. X513	NC_014538
Thermoanaerobacter tengcongensis MB4	NC_003869
Thermoanaerobacterium thermosaccharolyticum DSM 571	NC_014410
Thermobaculum terrenum ATCC BAA-798	NC_013525,NC_013526
Thermococcus sp. 4557	NC_015865
Thermocrinis albus DSM 14484	NC_013894
Thermodesulfatator indicus DSM 15286	NC_015681
Thermodesulfobacterium sp. OPB45	NC_015682
Thermodesulfobium narugense DSM 14796	NC_015499
Thermodesulfovibrio yellowstonii DSM 11347	NC_011296
Thermofilum pendens Hrk 5	NC_008696,NC_008698
Thermomicrobium roseum DSM 5159	NC_011959,NC_011961
Thermomonospora curvata DSM 43183	NC_013510
Thermoplasma acidophilum DSM 1728	NC_002578
Thermoproteus neutrophilus V24Sta	NC_010525
Thermosediminibacter oceani DSM 16646	NC_014377
Thermosipho africanus TCF52B	NC_011653
Thermosipho melanesiensis BI429	NC_009616
Thermosphaera aggregans DSM 11486	NC_014160
Thermosynechococcus elongatus BP-1	NC_004113
Thermotoga sp. RQ2	NC_010483
Thermotoga lettingae TMO	NC_009828
Thermotoga maritima MSB8	NC_000853
Thermotoga naphthophila RKU-10	NC_013642
Thermotoga neapolitana DSM 4359	NC_011978
Thermotoga petrophila RKU-1	NC_009486
Thermotoga thermarum DSM 5069	NC_015707
Thermovibrio ammonificans HB-1	NC_014917,NC_014926
Thermovirga lienii DSM 17291	NC_016148,NC_016149
Thermus scotoductus SA-01	NC_014974,NC_014975
Thioalkalimicrobium cyclicum ALM1	NC_015581
Thioalkalivibrio sp. K90mix	NC_013889,NC_013930
Thiobacillus denitrificans ATCC 25259	NC_007404
Thiomicrospira crunogena XCL-2	NC_007520
Thiomonas intermedia K12	NC_014153,NC_014154,NC_014155
Tolumonas auensis DSM 9187	NC_012691
Treponema azotonutricium ZAS-9	NC_015577

Reference organism	Accession numbers
Treponema brennaborense DSM 12168	NC_015500
Treponema denticola ATCC 35405	NC_002967
Treponema pallidum subsp. pallidum str. Nichols	NC_000919
Treponema pallidum subsp. pallidum SS14	NC_010741
Treponema paraluiscuniculi Cuniculi A	NC_015714
Treponema primitia ZAS-2	NC_015578
Treponema succinifaciens DSM 2489	NC_015385,NC_015386
Trichodesmium erythraeum IMS101	NC_008312
Tropheryma whipplei TW08/27	NC_004551
Truepera radiovictrix DSM 17093	NC_014221
Tsukamurella paurometabola DSM 20162	NC_014158,NC_014159
Uncultured_Arcobacter_1	1
Ureaplasma parvum serovar 3 str. ATCC 27815	NC_010503
Ureaplasma parvum serovar 3 str. ATCC 700970	NC_002162
Ureaplasma urealyticum serovar 10 str. ATCC 33699	NC_011374
Variovorax paradoxus EPS	NC_014931
Veillonella parvula DSM 2008	NC_013520
Verminephrobacter eiseniae EF01-2	NC_008771,NC_008786
Vibrio sp. Ex25	NC_013456,NC_013457
Vibrio anguillarum 775	NC_015633,NC_015637
Vulcanisaeta distributa DSM 14429	NC_014537
Waddlia chondrophila WSU 86-1044	NC_014225,NC_014226
Weeksella virosa DSM 16922	NC_015144
Weissella koreensis KACC 15510	NC_015756,NC_015759
Wigglesworthia glossinidia endosymbiont of Glossina brevipalpis	NC_003425,NC_004344
Wolbachia endosymbiont strain TRS of Brugia malayi	NC_006833
Wolinella succinogenes DSM 1740	NC_005090
Xanthobacter autotrophicus Py2	NC_009717,NC_009720
Xanthomonas albilineans GPE PC73	NC_013722
Xenorhabdus bovienii SS-2004	NC_013892
Xylella fastidiosa 9a5c	NC_002488,NC_002489,NC_002490
Yersinia enterocolitica subsp. enterocolitica 8081	NC_008791,NC_008800
Zobellia galactanivorans	NC_015844
Zunongwangia profunda SM-A87	NC_014041
Zymomonas mobilis subsp. mobilis NCIMB 11163	NC_013355,NC_013356,NC_013357,NC_013358
Nostoc azollae	NC_014248,NC_014249,NC_014250
Cyanobacterium UCYN-A	NC_013771
Gamma proteobacterium HdN1	NC_014366
Halophilic archaeon DL31	NC_015954,NC_015955,NC_015959

Reference organism	Accession numbers
Uncultured Termite group 1 bacterium phylotype Rs-D17	NS_000191,NS_000192,NS_000193,NS_000194
Uncultured methanogenic archaeon RC-I	NC_009464
